# Long-term potentiation in the anterior cingulate cortex and chronic pain

**DOI:** 10.1098/rstb.2013.0146

**Published:** 2014-01-05

**Authors:** Min Zhuo

**Affiliations:** 1 Center for Neuron and Disease, Frontier Institutes of Life Science, Science and Technology, Xi'an Jiaotong University, Xi'an 710049, People's Republic of China; 2 Department of Physiology, Faculty of Medicine, University of Toronto, Medical Science Building, Room no. 3342, 1 King's College Circle, Toronto, Ontario, CanadaM5S 1A8; 3 Department of Brain and Cognitive Sciences, College of Natural Sciences, Seoul National University, Seoul 151-747, Korea

**Keywords:** chronic pain, anterior cingulate cortex, long-term potentiation, analgesia, adenylyl cyclase, cyclic adenosine monophosphate

## Abstract

Glutamate is the primary excitatory transmitter of sensory transmission and perception in the central nervous system. Painful or noxious stimuli from the periphery ‘teach’ humans and animals to avoid potentially dangerous objects or environments, whereas tissue injury itself causes unnecessary chronic pain that can even last for long periods of time. Conventional pain medicines often fail to control chronic pain. Recent neurobiological studies suggest that synaptic plasticity taking place in sensory pathways, from spinal dorsal horn to cortical areas, contributes to chronic pain. Injuries trigger long-term potentiation of synaptic transmission in the spinal cord dorsal horn and anterior cingulate cortex, and such persistent potentiation does not require continuous neuronal activity from the periphery. At the synaptic level, potentiation of excitatory transmission caused by injuries may be mediated by the enhancement of glutamate release from presynaptic terminals and potentiated postsynaptic responses of AMPA receptors. Preventing, ‘erasing’ or reducing such potentiation may serve as a new mechanism to inhibit chronic pain in patients in the future.

## 1. Introduction

Pain and pleasure are two key factors in most parts of daily life. While we work to survive in a competitive society, and seek different forms of pleasure, we try to avoid pain when it is possible. The study of pain is mainly driven by humans' desire to avoid or control pain. The discovery of analgesic drugs such as opioids has greatly helped doctors to reduce the suffering of patients during and after surgery. However, while these drugs are effective for controlling pain which is short lasting (called acute pain), it is ineffective in controlling chronic pain. In addition, opioids have many unwanted side effects after prolonged use. Recent integrative neuroscience studies have found that chronic pain and acute pain operate through different central mechanisms [1]. Inhibitors that reduce pain transmission do not prevent the induction of chronic pain-related synaptic potentiation (called long-term potentiation, LTP), and inhibitors that abolish potentiation do not affect acute or physiological pain [1,2]. One key feature of chronic pain in the central nervous system is LTP triggered by peripheral injury and ongoing abnormal sensory inputs afterwards.

LTP, the primary experimental model for studying the synaptic basis for learning and memory [3,4], has been reported in sensory pain-related central synapses such as spinal dorsal horn and cortical areas that are important for pain perception [1,2,5]. In the past 10 years or so, some key progress has been made in understanding the molecular mechanisms of pain-related LTP, and its relevance to chronic neuropathic and inflammatory pain. More importantly, the mechanisms of the induction and expression of pain-related LTP potentially provide new therapeutic targets for controlling chronic pain in patients. In this review, I will focus on recent work on LTP in pain-related cortical areas, mainly the anterior cingulate cortex (ACC).

## 2. Pain and cortex

**Figure 1.**
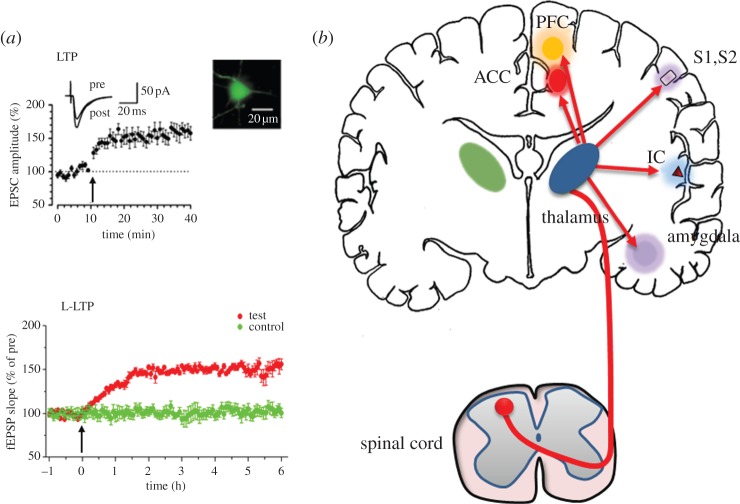
Long-term potentiation and chronic pain. (*a*) Neurons in the anterior cingulate cortex (ACC) are believed to play critical roles in pain perception and chronic pain. In ACC pyramidal cells (see inset image for an example), pairing presynaptic stimulation with postsynaptic depolarization induces long-term increases in the amplitudes of AMPA receptor-mediated excitatory postsynaptic currents (EPSCs). Similar potentiation can be induced by theta burst stimulation (TBS) and spike-timing LTP induction protocols (adapted from Zhao *et al.* [6]). In the lower plot, it shows that strong TBS induced late phase LTP (L-LTP) in the ACC (red circles), and control pathways show stable responses over the same period of time (green circles). (*b*) Under physiological conditions, noxious stimuli activate nociceptive afferent fibres (A*_*δ*_* and C fibres). Incoming action potentials trigger a release of excitatory transmitter glutamate in the spinal dorsal horn and neuropeptides including substance P (SP) and neurokinin A (NKA). Glutamate and neuropeptides activate spinal dorsal horn neurons, including those that send projection terminals to supraspinal structures. Neurons in the thalamus play key roles in relaying these ascending inputs. Several cortical areas are activated, including the ACC, prefrontal cortex (PFC), insular cortex (IC), primary somatosensory cortex (S1) and secondary somatosensory cortex (S2). Activation of the amygdala (as well as the ACC) also contributes to pain-related fear memory and pain modulation.

The study of pain benefits from the understanding of peripheral and spinal physiology. It has been known that selective sensory fibres and receptors are important for the initiation of pain transmission. Nerve activity caused by activation of nociceptors conveys to selective cortical regions that aid the formation of pain perception and unpleasantness of pain (figure 1). Cumulative evidence suggests that interfering with cortical activity can affect pain perception, including that in chronic pain conditions. Furthermore, pain-related cortical areas undergo plastic changes and can generate pain perception even without detectable sensory inputs from the periphery, such as the conditions of phantom pain, spontaneous pain in chronic pain conditions and the so-called central pain in different clinical disease conditions [1,7,8].

## 3. Pain perception is probably processed by a few key cortical areas

Among several cortical areas (figure 1), the ACC is a key cortical region for pain perception [1,7,9,10]. The ACC consists of different layers of pyramidal cells and local interneurons. Pyramidal cells are located in layers II, III and V. Anatomic and functional studies reveal that ACC pyramidal cells receive sensory inputs projecting from the thalamus as well as other subcortical structures. For the output projections of the ACC, pyramidal cells, especially those located in deep layer V, project to sensory related brain areas, including the motor cortex, amygdala, midbrain areas, brainstem and spinal cord [2]. *In vivo* electrophysiological recordings of neurons from animals as well as humans found that ACC neurons respond to peripheral noxious stimuli, and show increased responses to greater intensity of pain. This key finding is further supported by numerous human imaging studies in both normal individuals and patients with chronic pain. Human brain imaging studies further demonstrate that ACC neurons can be ‘activated’ in emotional pain conditions such as sadness due to loss of loved ones or emotional divorce. Animal behavioural studies using pharmacological agents find that inhibition of ACC activity is analgesic in different animal models of acute pain as well as chronic pain. Although studies using direct manipulations in human brains are limited, some clinical reports using electronic lesions or surgical ablation can reduce chronic pain caused by cancer [1]. Cognitive side effects have been reported in patients after the removal or lesions in the ACC, because the same region plays important roles in cognition and other executive functions. It is also worthwhile to note that deep brain stimulation will not provide any better selectivity of activation in possible clinical relief of chronic pain in patients. It is thus critical to identify selective or relatively selective cortical molecular targets that are involved in chronic pain to avoid cognitive side effects.

## 4. Long-term potentiation of excitatory sensory synapses may explain behavioural hyperalgesia and allodynia in chronic pain

**Figure 2.**
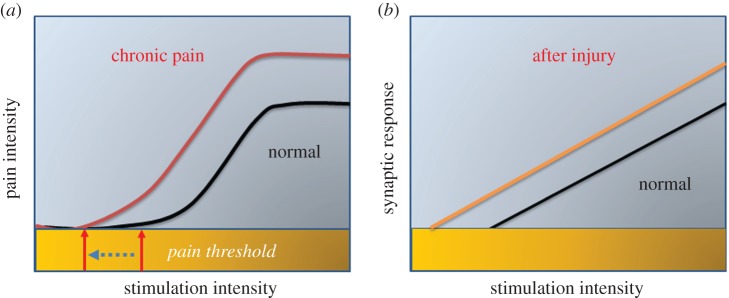
Synaptic potentiation as a cellular model for behavioural hyperalgesia and allodynia in chronic pain. (*a*) A model plot explains the hyperalgesia and allodynia in chronic pain conditions. Central plasticity causes the leftward shift of the input–pain response curve. The reduction of pain threshold into the non-noxious range possibly contributes at least partially to behavioural allodynia, whereas enhanced responses to the same noxious stimuli may contribute to hyperalgesia. (*b*) A model plot of synaptic responses versus stimulation intensity. Possibly, owing to changes in synaptic function (postsynaptic and presynaptic), the input–output curves shift leftward. These synaptic changes may contribute to behavioural hyperalgesia and allodynia at behavioural levels. Such changes may occur along sensory transmission pathways, from peripheral nociceptors, spinal dorsal horn synapses, thalamic neurons as well as cortical cells.

Hyperalgesia and allodynia are two major forms of sensitized behavioural responses measured in the animal models of chronic pain, such as inflammatory pain and neuropathic pain. In the case of hyperalgesia, nociceptive responses to noxious stimuli are significantly enhanced, and in allodynia, previously non-noxious stimuli cause behavioural nociceptive responses such as cold stimuli or non-noxious mechanical touch or brush (figure 2). At the cellular or synaptic level, hyperalgesia and allodynia may be mimicked by changes in synaptic transmission in spinal cord dorsal horn or cortical synapses. For hyperalgesia, enhanced synaptic responses, mainly by postsynaptic modification of AMPA (2-amino-3-(3-hydroxy-5-methyl-isoxazol-4-yl) propanoic acid) receptors, may directly contribute to enhanced sensory responses to stimuli. In the case of allodynia, the recruitment of ‘silent’ synapses, or increases in synaptic AMPA receptors may explain the activation of nociceptive neurons by subthreshold sensory stimulation.

Differing from behavioural allodynia and hyperalgesia, spontaneous pain has been less frequently investigated. This is because of the lack of appropriate behavioural models. Recent studies showed that animal place avoidance test can be used to evaluate spontaneous pain or tonic pain [11,12]. It is likely that neuronal correlates for spontaneous pain may be spike activities from pain or pain-related memory loci.

## 5. Pain-related spinal long-term potentiation and heterosynaptic facilitation

Studies suggest LTP in spinal dorsal horn neurons is believed to be the key mechanism for potentiation of sensory transmission after injury. Previous electrophysiological studies consistently indicated that spinal responses to sensory stimuli including non-noxious stimuli are enhanced. Electrical stimulation of sensory fibres or peripheral injury indeed induced LTP-like enhancement in spinal dorsal horn neurons [5]. LTP happens only in some spinal projecting cells [13]. Dorsal horn neurons that do not express substance P (SP) receptors do not undergo potentiation. Furthermore, activation of NK1 receptors or NMDA (*N*-methyl-d-aspartate) receptors is required for LTP [13]. Using the typical pairing protocol, Wei *et al*. [14] reported that LTP can be induced in dorsal horn neurons of adult mice. It is quite likely that different forms of LTP may exist in spinal dorsal horn synapses.

In addition, the activation of NMDA receptors and application of serotonin (5-HT) or 5-HT receptor agonists induced long-term facilitation of synaptic response [15,16], a heterosynaptic form of LTP. One mechanism for the facilitation is the recruitment of silent synapses through interaction of glutamate AMPA receptors with proteins containing postsynaptic density-95/discs large/zona occludens-1 (PDZ) domains [17]. Postsynaptic injection of a synthetic peptide corresponding to the last 10 amino acids of GluR2 (‘GluR2-SVKI’: NVYGIESVKI) that disrupts binding of GluA2 (previously called GluR2) to GRIP [16] blocked the facilitatory effect of 5-HT. The effect of GluA2-SVKI on synaptic facilitation is rather selective, because baseline excitatory postsynaptic currents (EPSCs) and currents evoked by glutamate application did not change over time in these neurons [16]. Experiments with different control peptides consistently indicate that the interaction between the C-terminus of GluA2/3 and GRIP/ABP (called GRIP1 and GRIP2) is important for 5-HT-induced facilitation.

## 6. Pain-related cortical synapses are plastic

It is commonly thought that mature cortical synapses are less plastic. In certain cortical areas, LTP is found to be age-dependent under experimental conditions [18]. The disappearance of cortical LTP is related to the disappearance of glutamate silent synapses or maturation of cortical synapses. In ACC synapses, the pattern of the induction protocols used for triggering LTP is important (table 1). High-frequency stimulation, a typical LTP induction protocol in the hippocampus, does not cause reliable long-lasting potentiation in the ACC [17]. However, theta burst stimulation (TBS), another learning-related LTP induction protocol, can induce LTP in both ACC synapses of young and adult animals (table 1). Furthermore, TBS-like neuronal activity has been reported from *in vivo* recordings of freely moving mice during trace fear learning, indicating that the TBS protocol mimics physiological conditions in the ACC [19]. In addition, LTP can also be induced using two other protocols, including the pairing training protocol and the spike–excitatory postsynaptic potential (EPSP) pairing protocol [20].

**Table 1.** Different forms of LTP recorded in the ACC under experimental conditions.

	induction protocol	recording method	pharmacological inhibitor or gene manipulation
NMDAR-dependent	TBS pairing protocol; spike timing	field EPSP; whole-cell patch-clamp	AP-5; NR2B antagonist, NR2A antagonist
L-VGCCs-dependent	TBS	field EPSP	nimodipine
pre-LTP	pre-conditioning	field EPSP	KAINATE receptor antagonist
L-LTP	multiple TBS	field EPSP	anisomycin; antinomycin

## 7. Different forms of anterior cingulate cortex long-term potentiation

**Figure 3.**
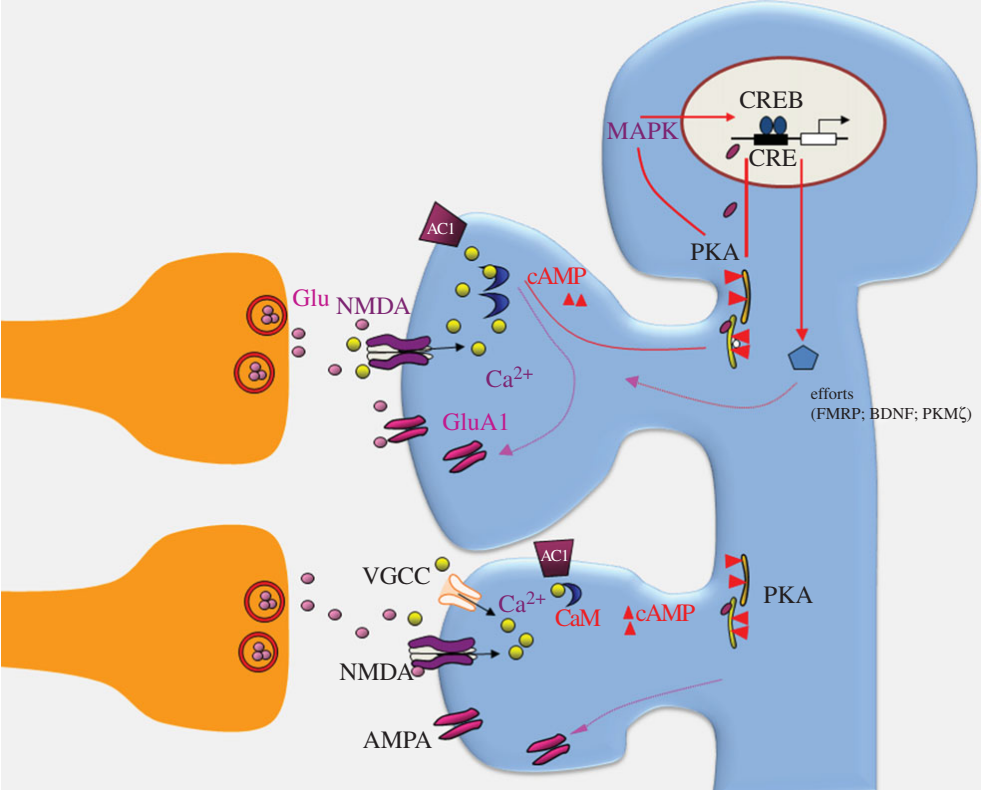
A synaptic model for LTP in the ACC. Activation of glutamate NMDA receptors leads to an increase in postsynaptic Ca^2+^ in dendritic spines. Both NMDA receptor containing GluN2B (NR2B) and GluN2A (NR2A) subunits are important for NMDA receptor functions in the ACC neurons. Ca^2+^ serves as an important intracellular signal for triggering a series of biochemical events that contribute to the expression of LTP. Ca^2+^ binds to calmodulin (CaM) and leads to activation of Ca^2+^-stimulated adenylyl cyclases (ACs), mainly AC1 and Ca^2+^/CaM-dependent protein kinases. The trafficking of postsynaptic GluA1 containing AMPA receptor contributes to enhanced synaptic responses. An NMDA receptor independent form of LTP can be also induced. Activation of L-type voltage-gated calcium channels (L-VGCCs) is important. It is likely similar calcium-dependent downstream signalling pathways are involved. In addition, activation of AC1 leads to activation of PKA-CREB, including MAP kinase (MAPK). Several candidate gene products may contribute to L-LTP, such as FMRP, BDNF and PKM*ζ*.

Pharmacological studied using selective receptor antagonists reveal that ACC LTP exists in at least four different forms: NMDA receptor-dependent, L-type voltage-gated calcium channel (L-VGCC)-dependent, late-phase LTP (L-LTP) and presynaptic LTP (pre-LTP) under experimental *in vitro* brain slice conditions (figure 3). Some of these LTPs may coexist under *in vivo* physiological or pathological conditions such as chronic pain or pathological fear memory (see below).

### (a) NMDA-receptor-dependent long-term potentiation

NMDA-receptor-dependent LTP has been commonly reported in central synapses [3,21]. In ACC synapses, LTP induced by different protocols is sensitive to the inhibition of NMDA receptors. Application of the NMDA receptor antagonist AP-5 blocked the induction of LTP. NMDA receptors containing GluN2A (called NR2A) or GluN2B (NR2B) subunits contribute to most of the NMDA receptor currents, and most of the GluN2B receptors are detected within synapses in ACC neurons [20]. Both application of the GluN2A antagonist NVP-AAM077 and of GluN2B antagonist ifenprodil or Ro 25-6981 produce an almost complete blockade of NMDA-receptor-mediated EPSCs. Application of GluN2A or GluN2B antagonist also reduces LTP, without complete abolishment of LTP. LTP is abolished only after the co-application of both inhibitors [20]. Interestingly, LTP induced by the spike-timing protocol seems to be more sensitive to NMDA GluN2B blockade as compared with effects on LTP induced by the pairing training protocol [20], suggesting the contribution of different subtype receptors may depend on the LTP induction protocol.

### (b) Calcium channel-dependent long-term potentiation

L-VGCC-dependent LTP has been reported in the hippocampus [21]. L-VGCCs are also required for the induction of LTP [22] by TBS in the field recording condition. Unlike LTP recorded using field recording, LTP recorded using whole-cell patch clamp does not respond to the inhibition of L-VGCCs. The exact mechanism for such difference remains to be investigated.

### (c) Protein synthesis-dependent late-phase long-term potentiation

Recent studies using a 64-channel multi-electrode array (MED64) show that ACC LTP induced by multiple TBS can last more than 5 h [12]. This form of potentiation is sensitive to inhibition of protein synthesis (Chen *et al.* 2014, unpublished data; see [23] for the insular cortex), indicating that protein synthesis-dependent L-LTP exists in the ACC. It is likely that L-LTP may contribute to long-term changes in the cortical circuits that are triggered by peripheral injury [12]. Future investigations of basic mechanisms are clearly required for ACC L-LTP.

### (d) Presynaptic form of long-term potentiation

To date, most forms of LTP reported in the ACC are postsynaptically induced. Our recent study reported that a new pairing protocol can induce LTP in the ACC that is purely presynaptically induced [24,25]. This presynaptic form of LTP (called pre-LTP) is completely independent of activation of postsynaptic NMDA receptors, and does not require calcium-dependent signalling pathways at postsynaptic sites.

## 8. Intracellular mechanisms for the induction of anterior cingulate cortex long-term potentiation

### (a) Postsynaptic calcium and calmodulin

Ca^2+^ serves as an important intracellular signal for triggering a series of biochemical events that contribute to the induction of ACC LTP. Postsynaptic injection of BAPTA into ACC neurons completely blocked the induction of LTP, indicating the importance of elevated postsynaptic Ca^2+^ concentrations [20]. Studies using electroporation of mutant calmodulin (CaM) in the ACC suggest that calcium binding sites of CaM_12_ are critical for the induction of cingulate LTP [26].

### (b) Calcium stimulated adenylyl cyclase subtype 1

Cyclic adenosine monophosphate (cAMP), produced by adenylyl cyclases (ACs), is a key second messenger in neural systems. Among more than 10 subunits, AC1 is the key AC subtype that responds positively to calcium–CaM [27]. Within the ACC, AC1 is highly expressed in cingulate neurons located in most layers [28]. AC1 is selective for plastic changes; gene deletion of AC1 does not affect basal glutamate transmission in the ACC. By contrast, LTP induced by TBS or pairing stimulation is abolished in cingulate pyramidal cells [22]. Whole-cell patch-clamp recording also reveals that AC1 activity is required for the induction of LTP in ACC pyramidal cells. By using chemical design and biochemical screening, several selective inhibitors of AC1 have been identified. Consistently, pharmacological inhibition of AC1 using the selective AC1 inhibitor NB001 abolished LTP in the ACC [29].

### (c) CaMKIV

Activity-dependent activation of immediate early genes such as cAMP response element-binding protein (CREB) is critical for synaptic plasticity [30,31]. A major neuronal signalling pathway by which Ca^2+^ activates CREB involves the Ca^2+^/CaM-dependent protein kinase type IV (CaMKIV). CaMKIV is distinguished among the CaM kinases in its capacity to activate CREB-dependent transcription both by virtue of its nuclear localization and catalysis of CREB phosphorylation on serine 133. CaMKIV is enriched in the ACC [32], and genetic deletion of CaMKIV abolishes CaM translocation and subsequent CREB phosphorylation and activation. Synaptic potentiation induced by TBS was reduced or abolished in the ACC of CaMKIV knockout mice [32]. The requirement of CaMKIV for ACC LTP is further supported by the observation of enhanced LTP in CaMKIV genetically overexpressed mice [33].

### (d) Extracellular signal-regulated kinase

The extracellular signal-regulated kinase (ERK) in the mitogen-activated protein kinase (MAPK) cascade has been implicated in synaptic plasticity and is known to be activated in ACC neurons after injury [34]. In the ACC, application of P42/P44 MAPK inhibitors, PD98059 and U0126, suppressed the induction of cingulate LTP [35]. By contrast, the same inhibitors had no effect on the maintenance of cingulate LTP. Inhibitors of c-Jun N-terminal kinase (JNK) and p38, other members of the MAPK family, SP600125 and SB203850, also suppressed the induction of cingulate LTP.

## 9. Gene expression and synaptic potentiation

Under different physiological and pathological conditions, including fear conditioning, peripheral inflammation or nerve injury, several key immediate early genes and their related products are activated in the ACC neurons. They include CREB, Egr1 and fragile X mental retardation protein (FMRP). It is likely that they mainly contribute to L-LTP, although their possible roles in LTP of the first hour after induction cannot be completely ruled out.

### (a) CREB

In the ACC, CREB is activated in an activity-dependent manner, and is downstream from key intracellular messengers that are important for ACC LTP, such as AC1 and CaMKIV [1]. Genetic manipulations that affect ACC LTP also affect the expression of CREB under different conditions [28,32]. Recent studies of mice with overexpressed CREB allowed us to examine the possible contribution of CREB to ACC LTP [33]. Enhanced CREB activity enhanced ACC potentiation induced by TBS. Interestingly, early potentiation is also enhanced, suggesting possible early contribution of CREB to synaptic potentiation in the ACC.

### (b) Egr1

The zinc finger transcription factor Egr1 (also called NGFI-A, Krox24 or zif/268) is critical for coupling extracellular signals to changes in cellular gene expression. The upstream promoter region of the Egr1 gene contains binding sites for cyclic AMP-response elements (CREs), suggesting that Egr1 may act downstream from the CREB pathway. In ACC neurons, Egr1 activity is triggered by injury including peripheral inflammation or digit amputation [36]. One possible role of Egr1 is to contribute to synaptic potentiation. Mice with genetic deletion of Egr1 indeed shows deficits in ACC LTP. The defect in LTP is relatively selective, because basal excitatory synaptic transmission is unaffected [37].

### (c) Fragile X mental retardation protein

FMRP is a ubiquitously expressed mRNA binding protein associated with polyribosomes and is thought to be involved in the translational efficiency and trafficking of certain mRNAs [38,39]. FMRP is predominantly a cytoplasmatic protein, but it does shuttle between the nucleus and cytoplasm, and transports selective mRNA molecules to their final destination within the cell. It is believed that FMRP may play roles in local protein synthesis that may be also required for LTP. Using FMR1 knockout mice, we found that ACC LTP was abolished in these mice [40]. The possible interaction between CREB and FMRP has been examined using genetically manipulated mice. In CREB overexpression mice, the increases of FMRP triggered by activation of metabotropic glutamate receptors are significantly enhanced [6], whereas in FMRP knockout mice, activation of CREB is similar to wild-type mice. These results suggest that FMRP is likely to act downstream from CREB in ACC neurons. Direct evidence for their relationship in LTP remained to be investigated. In addition to the induction of LTP, FMRP also plays roles in dopamine-induced enhancement of LTP [41]. FMRP thus acts as a key messenger for dopamine-produced modulation in the forebrain neurons [42].

## 10. Postsynaptic AMPA GluA1 receptor is critical for the expression of anterior cingulate cortex long-term potentiation

A major form of LTP in the ACC requires activation of postsynaptic NMDA receptors. The expression of ACC LTP is likely mediated by postsynaptic modification/trafficking of AMPA receptors. The roles of GluA1 (or called GluR1 or GluRA) and GluA2/3 (or called GluR2/3 or GluRB or C) in ACC LTP have been investigated using genetic and pharmacological approaches. Postsynaptic injection of GluA1 subunit C-terminal peptide analogue Pep1-TGL blocked cingulate LTP [43], indicating that the interaction between the C-terminus of GluA1 and PDZ domain proteins is required for ACC LTP. Furthermore, pharmacological experiments show that the application of philanthotoxin-433 (PhTx) 5 min after paired training reduces synaptic potentiation, whereas PhTx had no effect on basal responses. These results suggest that Ca^2+^-permeable GluA2-lacking receptors contribute to the maintenance of LTP and are necessary for subsequent LTP stabilization. GluA2/3 subunits may continually replace synaptic GluA2/3 subunits in an activity-independent manner that maintains constant synaptic transmission. We also examined the role of these peptides in synaptic potentiation in the ACC and found that the GluA2/3–PDZ interaction had no effect on cingulate LTP. We did find that the same interfering peptides inhibited cingulate long-term depression (LTD) [43]. These findings suggest that GluA1 and GluA2/3 play different roles in cingulate LTP versus LTD. The requirement of AMPA GluA1 receptor is further supported by data from gene knockout mice. In GluA1 knockout mice, ACC LTP is abolished. However, LTP is intact or slightly enhanced in mice lacking GluA2 [44].

## 11. Protein kinase M zeta activity is required for the maintenance of anterior cingulate cortex long-term potentiation

**Figure 4.**
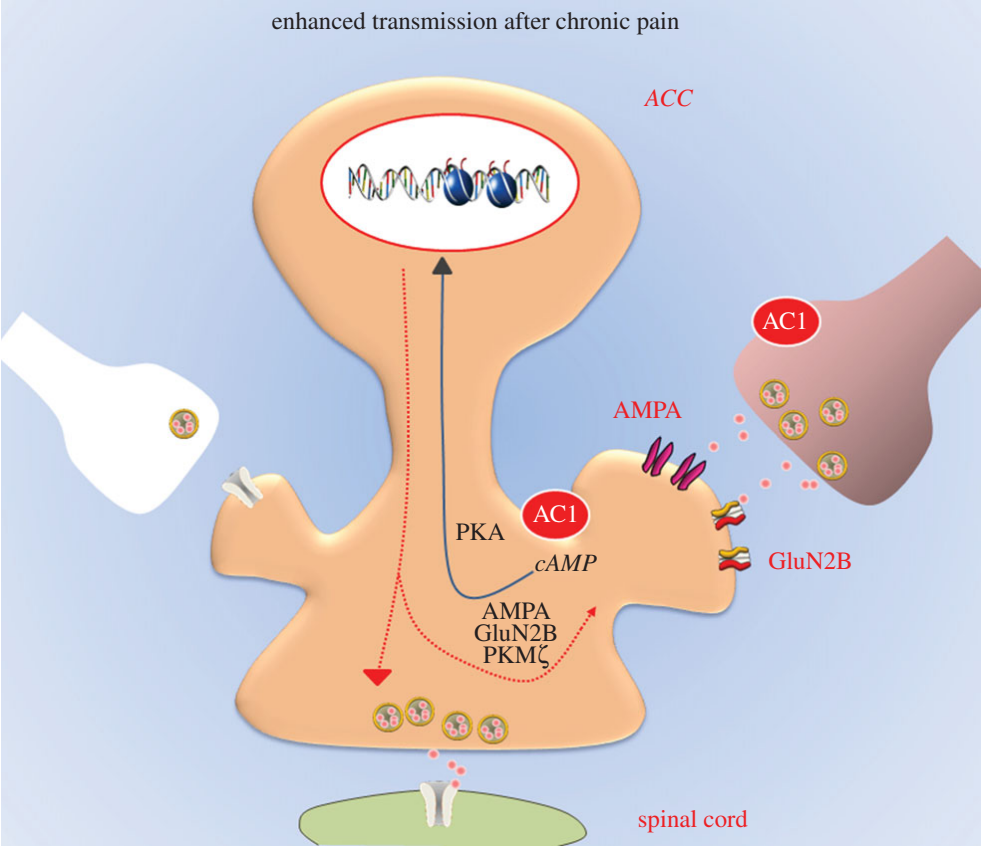
Postsynaptic and presynaptic mechanisms of chronic pain in the cortex. Excitatory synaptic transmission (left). Action potentials from the thalamic projection fibres trigger release of vesicles containing glutamate. Fast excitatory transmission is mediated by glutamate in the ACC. EPSCs are mediated by AMPA receptors, while small percentages of EPSCs are mediated by kainate receptors. Peripheral inflammatory or nerve injuries lead to presynaptic and postsynaptic changes within ACC synapses (right). At postsynaptic sites, the insertion of additional AMPA receptors and NMDA GluN2B (NR2B) receptors into active synapses significantly enhance synaptic responses to presynaptic stimulation. Similar to ACC LTP (figure 3), AC1-dependent PKA and CREB pathways are probably important for triggering and maintaining postsynaptic potentiation. In addition to postsynaptic potentiation, presynaptic enhancement of glutamate release also takes place after peripheral injury. Possible presynaptic activity of AC1 is critical for presynaptic potentiation. The exact mechanism for injury triggering presynaptic enhancement remains to be elicited in future studies. Enhanced excitability of neurons in the ACC may also affect spinal nociceptive transmission by activating descending facilitatory system.

Protein kinase M zeta (PKMζ) has been suggested as a key factor in maintaining LTP in the learning-related hippocampus [45,46]. In the ACC, recent studies indicate that PKMζ is important for maintaining L-LTP. Blocking the activities of PKMζ by ζ-pseudo-substrate inhibitory peptide (ZIP) erased late-phase LTP induced by TBS (figure 4) [12]. By contrast, ZIP alone did not affect baseline synaptic responses in the ACC. Similar effects of ZIP on L-LTP have been found in the insular cortex, another important brain area for chronic pain [23]. Therefore, PKMζ may interact with GluA1 to maintain L-LTP in the ACC. Inhibiting PKMζ activity can thus be potentially useful for treating pain [47,48].

## 12. *In vivo* anterior cingulate cortex long-term potentiation occurs in the anterior cingulate cortex after injury

In the brain slices, cingulate synapses can undergo LTP after the experimentally designed training protocols. One major question for *in vitro* study of LTP is its physiological or pathological relevance. One reason for studying ACC plasticity is its potential link to the injury-related central plasticity. Can LTP be detected *in vivo* in the ACC after peripheral injury? Indeed, peripheral digit amputation triggers significant and long-lasting enhancement of synaptic responses within the ACC [49]. Importantly, under *in vivo* conditions, peripheral sensory activity is not important for potentiation, because a local anaesthetic, QX-314, injected into the hindpaw (5%) did not affect the synaptic potentiation in the ACC [49].

Because it is difficult to determine the origin of electrical changes using field recordings *in vivo*, we performed intracellular recordings from anaesthetized rats. We found that there was a long-lasting membrane potential depolarization in ACC neurons of adult rats after digit amputation *in vivo* [50]. Shortly after digit amputation of the hindpaw, the membrane potential of intracellularly recorded ACC neurons quickly depolarized from about −70 to −15 mV and then slowly repolarized. The duration of this amputation-induced depolarization was about 40 min. Intracellular staining revealed that these neurons were pyramidal neurons in the ACC. An NMDA receptor antagonist, MK-801, significantly reduced the depolarization. Furthermore, the evoked EPSPs from ACC pyramidal cells to peripheral sensory stimuli were increased significantly and lasted for up to 7 days [51].

## 13. Detection of anterior cingulate cortex potentiation in *in vitro* brain slices after injury

Although *in vivo* electrophysiology provides direct evidence for pathological relevance of LTP in the ACC, it is difficult for studying cellular and molecular mechanisms. Detecting synaptic changes in the brain slices of animals with chronic pain will provide us opportunities to study the basic mechanisms of chronic pain. In fact, both postsynaptic and presynaptic changes are reported in the brain slices of animals with inflammatory or neuropathic pain. Furthermore, in addition to changes in glutamate AMPA receptors, NMDA GluN2B receptors are also found to undergo LTP.

### (a) AMPA receptors

To detect possible changes in synaptic transmission within the ACC after nerve injury, AMPA-receptor-mediated EPSCs in pyramidal neurons in the layer II/III of the ACC were measured in mice with peripheral nerve ligation [52]. The input (stimulation intensity)–output (EPSC amplitude) curve of AMPA-receptor-mediated current was significantly shifted to the left after peripheral nerve injury, compared with that in control group. These results suggest that excitatory synaptic transmission is increased in the ACC after peripheral nerve injury. Similar changes are found in ACC neurons in animal models of inflammation induced by complete Freund's adjuvant (CFA) [53]. Enhanced synaptic transmission is also found in rats with inflammation. Bie *et al.* [54] reported that CFA inflammation in rats triggered increased AMPA-receptor-mediated responses in ACC neurons with observed leftward shift of the input–output curves. In addition, the rectification of AMPA-receptor-mediated transmission in the ACC between control and nerve-ligated mice is different [52]. These results demonstrate that AMPA receptor in ACC neurons has an inward rectification property in neuropathic pain. Similar rectification of the AMPA-receptor-mediated responses in ACC neurons of rats has been reported after peripheral inflammation with hindpaw CFA injection.

Biochemical and anatomic evidence further support the involvement of postsynaptic AMPA receptors [55]. The membrane AMPA GluA1 receptors are significantly increased, whereas GluA2/3 receptors are not significantly affected. Such increases are dependent on PKA phosphorylation of GluR1 receptors. Recent electronic microscope data further support that postsynaptic membrane GluA1 is significantly increased after peripheral injury (Chen *et al.* 2014, unpublished data).

### (b) NMDA GluN2B receptors

Previous studies using genetically manipulated NR2B (or GluN2B) mice found that chronic pain is significantly enhanced in transgenic mice [56–58]. Does genetic overexpression of GluN2B mimic physiological or pathological conditions? Electrophysiological and biochemical studies consistently indicate that peripheral injuries indeed cause the increases of NMDA GluN2B receptors in the ACC (as well as the insular cortex). After nerve injury or inflammation, cortical NMDA GluN2B receptors are significantly enhanced [50,59]. Furthermore, the upregulation of NMDA GluN2B receptors in the ACC or insular cortex contributes to inflammation- or nerve-injury-related persistent pain [50] (figure 4). In the case of persistent inflammation, the expression of NMDA GluN2B receptors in the ACC is upregulated, thereby increasing the GluN2B component in NMDA-mediated responses [60]. Consistently, microinjection into the ACC or systemic administration of GluN2B receptor selective antagonists inhibits behavioural responses to peripheral inflammation. Recent studies in the insular cortex reveal that nerve injury also triggers the upregulation of NMDA GluN2B receptors [59]. Thus, the central NMDA GluN2B receptor is a potential drug target for controlling chronic pain [58,61,62].

### (c) Presynaptic release

In addition to postsynaptic enhancement, recent studies indicate that presynaptic release of transmitter is also enhanced in the ACC [63]. Paired-pulse facilitation (PPF) is a transient form of plasticity commonly used as a measure of presynaptic function, in which the response to the second stimulus is enhanced as a result of residual calcium in the presynaptic terminal after the first stimulus. After nerve ligation, there was a significant reduction in PPF in ACC neurons compared with those from control mice. These results indicate that there is presynaptic enhancement of the excitatory synaptic transmission in the ACC after nerve injury [52]. Similar changes in PPF ratio are found in ACC neurons of animals with CFA inflammation [40], indicating that presynaptic enhancement of glutamate release is also shared by peripheral inflammation.

In addition to the use of PPF in the ACC after peripheral nerve injury, AMPA-receptor-mediated miniature EPSCs (mEPSCs) in ACC neurons in the presence of 0.5 μM tetrodotoxin were also found to be affected. After peripheral nerve injury, there was an obvious increase of mEPSC frequency in ACC neurons compared with that of control groups. Considering most LTP studied in the ACC is postsynaptically expressed, it is important to develop an experimentally induced pre-LTP protocol to mimic presynaptic changes of glutamate release in the ACC after peripheral injury.

## 14. AC1 as a potential target for blocking long-term potentiation and treatment of chronic pain

**Figure 5.**
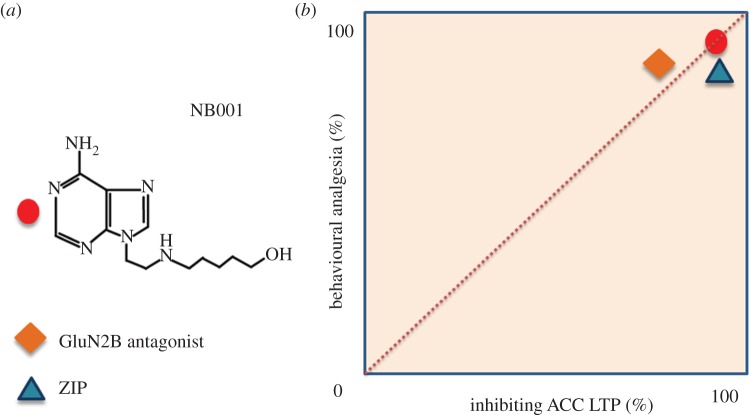
Translational investigation of novel drug targets those are required for pain plasticity in chronic pain. (*a*) The chemical structure of a novel inhibitor for AC1, NB001. (*b*) Drugs that inhibit cortical LTP also show powerful analgesic effects in animal models of chronic pain including chronic inflammatory pain and neuropathic pain. These include inhibitors that prevent the induction of ACC LTP such as NB001 and NMDA GluNR2B (NR2B) receptor antagonists as well as inhibitor that erase ACC LTP such as PKM*ζ* inhibitor ZIP.

Among several key second messengers that contribute to ACC LTP, AC1 is unique for LTP in this region. Previous studies using AC1 knockout mice as well as recent pharmacological studies of the AC1 inhibitor NB001 consistently showed that AC1 or AC1 activity is not essential for hippocampus LTP and behavioural learning [29,55] (figure 5). By contrast, AC1 plays a critical role in pain-related LTP in both the spinal cord dorsal horn and ACC [22,29]. Behaviourally, AC1 knockout mice show reduced inflammatory, deep muscle pain and neuropathic pain [55], whereas other physiological functions remain intact in AC1 knockout mice, including acute pain, Morris water maze performance, as well as anxiety-like behaviours and motor functions. Considering that AC1 is mainly expressed in the central nervous system [27], AC1 is proposed to be a suitable neuron-specific drug target for treating neuropathic pain. Using rational drug design and chemical screening, we have identified a lead candidate AC1 inhibitor, NB001, which is relatively selective for AC1 over all other AC isoforms. Using a variety of behavioural tests and toxicity studies, we have found that NB001, when administered intraperitoneally or orally, has an analgesic effect in animal models of neuropathic pain, without any apparent side effects [29]. These findings show that AC1 could be a productive therapeutic target for neuropathic pain and chronic inflammatory pain. Considering the fact that many signalling molecules that are important for ACC LTP overlap with those for hippocampal LTP, it is important to note that AC1 is unique in playing essential roles in different forms of ACC LTP but not in hippocampal LTP. Future investigations of LTP mechanisms in these two regions may provide new drug targets for treating chronic pain with less cognitive side effects.

In addition to producing analgesic effects by inhibiting the upregulation of AMPA receptors in chronic pain conditions, our recent studies found that AC1 activity is also critical for the upregulation of NMDA GluN2B receptors in the insular cortex [59]. These findings provide strong evidence that AC1 plays dual roles in the chronic pain related to the upregulation of AMPA and NMDA receptors, further supporting AC1 as a novel target for treating chronic pain in the future [61,64]. Finally, previous reports found that presynaptic enhancement after nerve injury or inflammation is also blocked in mice lacking AC1, indicating that presynaptic AC1 is also important for presynaptic changes. Future studies are required to examine the mechanism for presynaptic changes.

## 15. Conclusion and future directions

From a traditional point of view, there are many good reasons for scientists and drug developers to avoid studying the ACC for the treatment of chronic pain. The spinal dorsal horn and periphery are still seen as ‘gold’ targets for controlling pain. However, in the case of chronic pain, more and more evidence indicates that some central changes occur right after the injury. Just like fear memory and other learning process, it is too late to interfere at the periphery if such ‘bad’ memory takes place in the brain. Many failed surgical manipulations for chronic pain patients prove the case. Many e-mails from chronic pain patients I have received indicate that the current medical care systems, modern medicines as well as alternative medicine, often fail to help. These individuals include patients with spinal cord injury, cancer, neuropathic pain, etc. So far among drugs approved by the US Food and Drug Administration for the treatment of pain, there is no single class of pain medicine that targets injury-related plasticity.

From a basic point of view, cumulative evidence demonstrates that pain or injuries activate important cortical areas such as the ACC, prefrontal cortex, insular cortex, amygdala and the hippocampus. The impact of such injury on emotion and cognitive functions is traditionally ignored. Here, I would like to propose four major possible research questions that I believe are highly relevant for ACC plasticity.

First, is LTP equally occurring in thalamic inputs and cortical–cortical connections (including connections between different layers of cells within the ACC, connections between ACC cells and other cortical areas)? Dual patch-clamp recordings show that cortical and cortical connections can even occur within local neurons located in different layers of cells within the ACC [65]. Previous studies in brain slices, as well as preliminary results *in vivo*, show that ACC–ACC synapses and thalamus–ACC synapses may both undergo LTP after training or injury [60]. It will be critical in the future to use *in vivo* approaches, and/or *in vitro* preparations if possible, to investigate if LTP in these two different pathways onto cingulate neurons may share similar mechanisms.

Second, is there any presynaptic form of ACC LTP? What are physiological or pathological functions of pre-LTP in the ACC? Electrophysiological and pharmacological studies reveal that there is presynaptic enhancement of glutamate releases in chronic inflammatory pain and neuropathic pain conditions. However, we also found that NMDA-receptor-dependent ACC LTP is purely postsynaptically expressed [40]. Thus, it is likely that different forms of LTP may exist in the ACC. New forms of LTP containing presynaptic components may remain to be discovered. Future experiments are clearly required to investigate these possibilities.

Third, what is the impact of synaptic LTP on neuronal coding within the ACC? One key question for synaptic plasticity is the impact of LTP on neuronal action potentials. Is LTP contributing to increases in neuronal firing to subsequent stimuli such as in the case of hyperalgesia and allodynia? Or does LTP contribute to special activity within a neuronal circuit?

Finally, what is the relationship between pain-induced plasticity and emotional disorders in chronic pain conditions? Depression, anxiety, loss of hope may be triggered by injury at the synaptic level. These ‘side effects’ become amplified if pain sensation persists owing to the failure of treatment or medicine. Is there any role of ACC LTP in pain-related emotion?

In summary, integrative approaches have yielded significant progress in our understanding of synaptic mechanisms for sensory transmission, modulation and plasticity in the ACC. The involvement of each signalling protein will be identified. The combination of different experimental approaches such as synaptic neurobiology, systems biology and neuronal circuits is clearly required to understand how synaptic plasticity affects human and animal behaviours under physiological and pathological conditions.
